# Quality of life in individuals initiating antiretroviral therapy: a cohort study

**DOI:** 10.11606/s1518-8787.2020054001920

**Published:** 2020-12-04

**Authors:** Gabriela Sales Pimentel, Maria das Graças Braga Ceccato, Juliana de Oliveira Costa, Jullye Campos Mendes, Palmira de Fátima Bonolo, Micheline Rosa Silveira

**Affiliations:** I Universidade Federal de Minas Gerais Faculdade de Farmácia Programa de Pós-Graduação em Medicamentos e Assistência Farmacêutica Belo HorizonteMG Brasil Universidade Federal de Minas Gerais. Faculdade de Farmácia. Programa de Pós-Graduação em Medicamentos e Assistência Farmacêutica. Belo Horizonte, MG, Brasil; II Universidade Federal de Minas Gerais Faculdade de Farmácia Departamento de Farmácia Social Belo HorizonteMG Brasil Universidade Federal de Minas Gerais. Faculdade de Farmácia. Departamento de Farmácia Social. Belo Horizonte, MG, Brasil; III University of New South Wales Centre for Big Data Research in Health Faculty of Medicine Sydney Australia University of New South Wales. Centre for Big Data Research in Health. Faculty of Medicine. Sydney, Australia; IV Universidade Federal de Minas Gerais Faculdade de Medicina Belo HorizonteMG Brasil Universidade Federal de Minas Gerais. Faculdade de Medicina. Pós-Graduação em Saúde Pública. Belo Horizonte, MG, Brasil; V Universidade Federal de Minas Gerais Faculdade de Medicina Departamento de Medicina Preventiva e Social Belo HorizonteMG Brasil Universidade Federal de Minas Gerais. Faculdade de Medicina. Departamento de Medicina Preventiva e Social. Belo Horizonte, MG, Brasil

**Keywords:** Anti-HIV Agents, Antiretroviral Therapy, Highly Active, Quality of Life, WHOQOLHIV-bref, Cohort Studies

## Abstract

**OBJECTIVE:**

To assess longitudinally the change in quality of life in people living with HIV initiating antiretroviral therapy in three public reference services specialized in HIV care in Belo Horizonte, Brazil.

**METHODS:**

Prospective cohort study among people living with HIV, aged 18 years or older, and initiating antiretroviral therapy. We obtained sociodemographic, behavioral, clinical data related to pharmacological treatment and to the service by face-to-face interviews, and supplemented these data with information from clinical records and Information Systems of the Brazilian HIV/AIDS Program. We measured the quality of life using the WHOQOL-HIV bref instrument, with a minimum interval of six months between the baseline and the follow-up interviews. We used paired t-test to assess the mean change in quality of life between the two interviewsand evaluated factors associated with this outcome using multiple linear regression.

**RESULTS:**

The overall quality of life, as well as the physical, psychological, level of independence, environment and spiritual quality of life domains were statistically higher in people living with HIV using antiretroviral therapy at the end of the follow-up. Factors independently associated with the increase in quality of life were having religious belief and living with other people. Having signs or symptoms of anxiety and depression and the number of adverse drug reactions reported were predictors associated with worsening quality of life.

**CONCLUSIONS:**

These results show an improvement in the quality of life over time in people living with HIV on antiretroviral therapy. They also highlight the need to monitor and provide health care support, especially for individuals with signs and symptoms of anxiety and depression and that report adverse reactions to medicines at the beginning of treatment.

## INTRODUCTION

In Brazil, in recent years, first-line antiretroviral (ARV) schemes have been modified, following the global trend and recommendations of the World Health Organization (WHO)^1^ . In 2015, the *Protocolo Clínico e Diretrizes Terapêuticas* (PCDT – Clinical Protocol and Therapeutic Guidelines) for the management of HIV recommended a fixed-dose combination (FDC) containing the medicines tenofovir (TDF), lamivudine (3TC) and efavirenz (EFV) as a first-line regimen. In 2017, dolutegravir (DTG), an integrase inhibitor, was recommended as the first-line regimen, replacing EFV. This medicine should be taken together with a fixed-dose combination of TDF and 3TC^2^ .

In developing countries, such as Brazil, evidence from longitudinal studies on the determinants of quality of life (QOL) in people living with HIV (PLWH) are scarce. In a literature review, gender, age, family situation, education, employment, income, viral load, TCD4+ lymphocyte count, diagnosis time, presence of symptoms of depression and anxiety, social support, health care, use of licit and illicit drugs, adherence to antiretroviral therapy (ART), lifestyle and sexual behavior were factors directly associated with PLWH QOL^3^ . Therefore, understanding QOL is essential to analyze the physical and biopsychosocial impact that HIV can cause, enabling greater knowledge of individuals about themselves, their adaptation to the condition of living with HIV and to their treatment. Evidence on the impact of ART initiation on QOL in PLWH is also scarce.

The objective of our study was to evaluate longitudinally the QOL in PLWH initiating ART that attended three public reference services in Belo Horizonte, Brazil. We further evaluated the predictors of change in QOL.

## METHODS

This is a cohort study, using data from the Ecoart project, whose details were previously published^4 , 5^ .

The sample selection was non-random, and all eligible individuals attending three public specialized HIV care services were invited to participate in the study. These servicesall together dispense ART for approximately 80% of PLWH in the municipality of Belo Horizonte, state of Minas Gerais,Brazil. Participants’ selection took place between September 2015 and October 2017.

We included individuals with up to 180 days of ART usage, who attended one of the services evaluated and who were identified through their registration in the logistic control system of medicines (Siclom), a national system of ART dispensation. The inclusion criteria were the signing of the informed consent form, being 18 years of age or older, autonomy to respond to the interview and having performed the baseline interview and the second follow-up interview.

We obtained data by face-to-face interviews and in secondary databases, using standardized forms that contained the World Health Organization quality of life HIV instrument – in its shortened version (WHOQOLHIV – bref), the Hospital scale of anxiety and depression (HADS) and the Morisky’s therapeutic adherence scale of eight items (MMAS–8), all validated in Brazil^6 - 8^ . It also contained questions regarding sociodemographic, behavioral, clinical, laboratory information, related to pharmacological treatment and to the health care service.

To minimize potential bias, researchers were properly trained and a pilot project was conducted before starting data collection.

### Dependent Variable

We assessed QOL using the WHOQOL-HIV bref instrument in two moments: at the baseline interview and at the second follow-up interview six months after the baseline interview. The mean difference in overall QOL between these two moments was the dependent variable of our study. Therefore, we present the results only of those individuals that concluded both interviews. Data on QOL at baseline were published previously^4^ .

The WHOQOL-HIV bref is a specific instrument for assessing QOL in PLWH, which, in 31 questions, evaluates the following domains: physical, psychological, level of independence, social relations, environment and spirituality. Each question has five-point Likert scale answer options, 1 indicating negative perceptions and 5, positive perceptions, except for seven items, in which the scale is reversed. The average of the items within each domain is used to estimate the total score for the domain. The result is multiplied by 4, and thus the scores vary between 4 and 20^6^ .

The overall QOL component and general health perception was evaluated using the first two questions of the WHOQOL-HIV bref instrument. The mean of the items was multiplied by 4; thus, the scores also ranged from 4 to 20^6^ .

### Independent Variables

We used sociodemographic, behavioral, clinical and laboratory variables related to pharmacological treatment and service to characterize the study population.

The presence of signs or symptoms of anxiety and depression was measured using the HADS, composed of 14 items^7^ . People that had 8 or more points in each of the conditions were classified with symptoms of anxiety and depression. The individuals were classified according to the presence of signs and symptoms of anxiety or depression in the baseline interview and in the follow-up interview, being categorized as: (I) never – they never presented signs and symptoms of anxiety or depression, (ii) ever – they presented these symptoms in one of the two interviews or (iii) always – they presented these symptoms in both interviews.

MMAS-8 was used to evaluate adherence to ART. The total score of MMAS-8 varies from 0 to 8, and the higher the score, the higher the adhesion^8^ . Individuals that scored 8 points were considered adherents. Adverse drug reactions (ADR) were assessed by self-reporting. Individuals with less than 50 copies per ml were considered with an undetectable viral load.

The variable economic class (socioeconomic status) was evaluated according to Brazilian criteria, such as high (A, B), intermediate (C) and low (D and E), in which individuals are classified by socioeconomic groups by possession of comfort items and level of education of the head of the family^9^ .

For the comorbidity variable, it was considered the self-report of any disease diagnosed by a physician, such as arterial hypertension, diabetes, hypercholesterolemia, among others.

### Statistical Analysis

We performed adescriptive analysis by frequency distribution for categorical variables and measures of central tendency for quantitative variables.

To evaluate the scores of the WHOQOL-HIV bref instrument, means and standard deviation were presented for each domain, relative to the baseline interview and the second follow-up interview. We then used paired *t* -test to compare the mean differences in QOL scores between the two interviews.

The univariate analysis of the relationship between independent variables and the change in QOL score was evaluated by the Student *t* test (comparison of means) and the Mann-Whitney test (comparison of medians).

We analyzed the association between change in QOL and independent variables by adjusting a multiple linear regression model. Variables with a p-value equal to or less than 0.20 were selected to enter the multivariate model. The backward stepwise method was used to obtain the final model. The results of multiple linear regression were demonstrated by regression coefficients, with their respective 95% confidence intervals (95%CI).

We evaluated the adequacy of the model by a set of statistics. The statistics R^2^ and adjusted R^2^ were used to verify the percentage of variance related to the increase in QOL explained by the model. The Durbin-Watson statistic was used to verify the assumption that the residues are not correlated, with values between 0 and 4, and 2 means no correlation between the residues. It was also tested if there was multicolinearity in the final model, using the statistics of tolerance (acceptable > 0.10) and VIF (acceptable < 10)^10^ . To evaluate if the residues had normal distribution, the following graphs were performed: standardized regression residues by standardized regression predicted values, histogram of frequencies of standardized regression residues, and a quantiles-quantile graph (QQ plot).

The Statistical Package for the Social Sciences (SPSS) software version 22.0 was used. In all analyses, a 0.05 significance level was considered.

### Ethical Aspects

The Ecoart research project was approved by the Research Ethics Committee (COEP) of the Universidade Federal de Minas Gerais (CAAE protocol 31192914.3.3001.5124, opinion CEP 769.085) and the participating services. The research was conducted following the instructions of resolution 466/2012 of the National Health Council.

## RESULTS

During the entire recruitment period of the Ecoart project, 1,017 individuals served in the three services were identified. Of these, 468 performed the baseline interview, of which 323 (69.0%) performed the second follow-up interview and were included in our study ( Figure 1).

**Figure:**
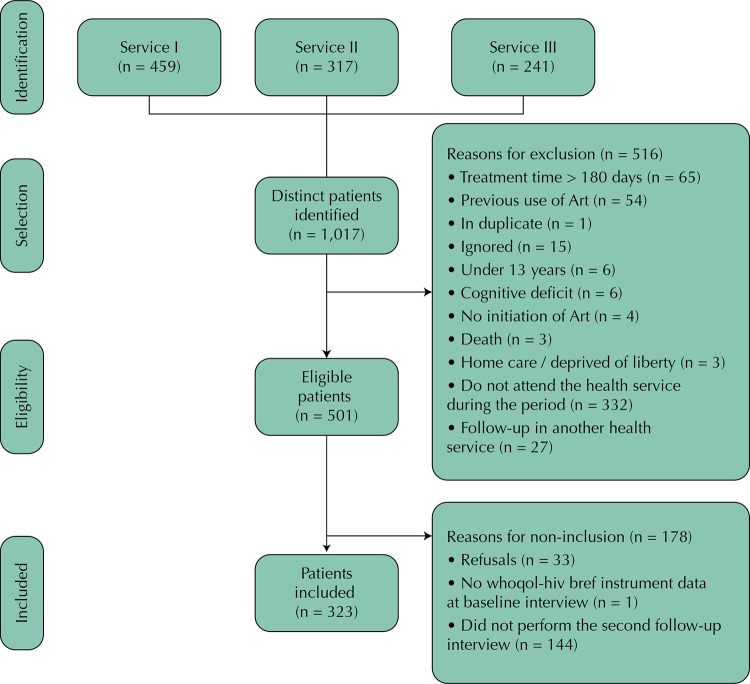
Diagram of individuals’ inclusion in the study.

No statistically significant differences regarding gender, age, marital status and education were found between the group that completed the second follow-up interview with those that did not. There were also no differences regarding general QOL and QOL domains.

Most of the respondents were men (83.0%), unmarried (79.9%), without children (66.9%), between 20 and 34 years old (54.5%), did not have a private health insurance (72.8%) and self-declared as non-white (76.9%). We found that 80.8% of the individuals lived with others, 38.8% had 10 to 12 years of schooling and 59.4% were employed at the time of the interview, with a predominance of Economy Class C (45.7%).

Regarding behavioral characteristics and lifestyle habits, 24.5% reported current tobacco use, 65.8% reported recent alcohol use, and 47.5% reported current or prior use of illicit drugs in their lives. Most reported having some religious belief (79.4%) and 59.6% were men who have sex with other men (MSM).

Regarding clinical characteristics, 7.8% presented coinfection (such as tuberculosis, syphilis, toxoplasmosis and candidiasis), 18.3%, one or more self-reported comorbidities, and 35.9 had signs or symptoms of anxiety and depression. Most individuals presented asymptomatic clinical classification (67.3%) and had less than or equal to six months of diagnosis of HIV infection (61.8 %). Regarding the baseline laboratory characteristics, 23.5% had an initial TCD4+ lymphocytes count less than 200 cells/mm^3^, 90.1%, detectable viral load, and 26.0%, viral load greater than 100,000 copies/ml.

Regarding pharmacological treatment, 63.2% used TDF/3TC/EFV, and 32.2%, TDF/3TC+DTG. Most (52.3%) had been using ART for 60 days or less, were non-adherent to ART (52.8%), reported at least one ADR (85.9%), and 53.1% reported three or less ADR ( Table 1 ).

**Table 1:** Sociodemographic, behavioral, clinical, laboratory, therapeutic and service-related characteristics of PLWH in initial ART in the baseline interview and in the second follow-up interview, Belo Horizonte, Minas Gerais (n = 323).

Baseline interview		
Sociodemographic	n	(%)
Characteristics		
Gender (male)	268	83
Age (years)		
18–19	10	3,1
20–34	176	54,5
35–49	102	31,6
≥ 50	35	10,8
Age (mean (SD)), years	34.5 (10.7)	
Marital Status (single/divorced/widow)	258	79,9
Skin color (non-white)	246	76,9
Schooling (years)		
≤ 9	75	23,3
10–12	125	38,8
≥ 13	122	37,9
Children (no)	216	66,9
Living with other people (yes)	261	80,8
Employed (yeas)	192	59,4
Health plan (no)	235	72,8
Economic status		
A, B	122	38,7
C	144	45,7
D, E	49	15,6
Behavioral		
Religious belief (yes)	255	79,4
Current smoking (yes)	79	24,5
Alcohol consumption in the last month (yes)	212	65,8
Use of illicit drugs in life (yes)	153	47,5
Risk / exposure category (MSM)	168	59,6
Clinical		
Clinical classification (asymptomatic)	214	67,3
Signs or symptoms of anxiety or depression (yes)	116	35,9
Self-reported comorbidities (no)	264	81,7
Self-reported co-infections (no)	296	92,2
HIV diagnosis time (mean (SD)), months	77.1 (59.8)	
Laboratorial		
TCD4 + lymphocyte count (cells / mm3)		
< 200	76	23.5
200–500	124	38.4
> 500	96	29.7
Missing data	27	8.4
VL*		
Detectable	291	90.1
Undetectable	5	1.5
Missing data	27	8.4
VL (copies/mL)		
≤ 100.000	212	65.6
> 100.000	84	26
Missing data	27	8.4
ART-related		
ART regimen		
TDF/3TC/EFV	204	63.2
TDF/3TC + DTG	104	32.2
Other regimens	15	4.6
ART treatment time (mean (SD)), days	77.1 (59.8)	
Adherence (no)	163	52.8
ADR (yes)	267	85.9
ADR frequency (mean (SD))	3.8 (3.2)	
Heath service-related		
Health care service		
I	106	32.8
II	144	44.6
III	73	22.6
Second follow-up interview		
Clinics		
Signs or symptoms of anxiety or depression		
Never (neither at baseline nor in the follow-up interview)	41	12.7
Ever (baseline or follow-up interview)	244	75.5
Always (baseline and follow-up interview)	36	11.1
Laboratorial		
VL (viral suppression)		
Detectable	43	13.3
Undetectable	237	73.4
Missing data	43	13.3
ART-Related		
Adherence (no)	191	59.5
ADR (yes)	165	51.6
ADR number (mean (SD))	1.9 (2.7)	

LT-CD4 +: T-CD4 + lymphocytes; MSM: men that have sex with men; TDF: tenofovir; 3TC: lamivudine; EFV: efavirenz; DTG: dolutegravir; ART: antiretroviral therapy; ADR: adverse drug reaction; HIV: human immunodeficiency virus; other risks: hemophiliacs, transfusion and occupational.

* Undetectable CV: < 50 copies/ml.

In the second follow-up interview, regarding clinical, laboratory and ART-related characteristics, 34.3% of individuals presented signs and symptoms of anxiety and depression, 73.4% undetectable viral load, 59.5% were non-adherent to treatment, and 51.6% reported some ADR, and 79.9% reported three or less ADR ( Table 1 ).

### QOL Results

The second follow-up interview occurred on average 9.47 months (median = 8.66; minimum = 3.5; maximum = 23.0; interquartile interval = 19.5, SD = 3.67) after the baseline interview.

Statistically significant improvements were observed over time in the overall QOL and in the physical, psychological, level of independence, environment and spiritual domains in individuals initiating ART. The only domain, in which the mean difference between baseline and follow-up interviews was not significant, was that of social relations.

The QOL domains with the greatest mean differences were physical (5.11; SD = -3.75), spiritual (3.23; SD = 6.20) and psychological (1.32; SD = 2.82) ( Table 2 ).

**Table 2:** Distribution of WHOQOL-HIV bref domain scores at baseline and after follow-up in individuals living with HIV on initial ART, Belo Horizonte, state of Minas Gerais (n = 323).

WHOQOL-HIV bref domains^a^	n	Baseline interview – mean (SD)	Second follow-up interview – mean (SD)	Mean difference between the second and the baseline interviews (SD)	p
Overall quality of life	323	15.37 (3.12)	16.24 (2.93)	0.86 (3.28)	< 0.001^b^
Physical	314	10.94 (2.20)	16.04 (2.94)	5.11 (3.75)	< 0.001^b^
Psychological	318	14.03 (2.06)	15.35 (2.73)	1.32 (2.82)	< 0.001^b^
Independence level	316	14.89 (2.26)	15.72 (2.86)	0.82 (3.08)	< 0.001^b^
Social relations	295	15.28 (3.01)	15.57 (2.83)	0.29 (3.15)	0.113
Environment	315	14.29 (2.40)	14.71 (2.36)	0.42 (2.08)	< 0.001^b^
Spiritual	315	11.67 (3.42)	14.89 (3.73)	3.23 (6.20)	< 0.001^b^

SD: standard deviation; ART: antiretroviral therapy.

^a^ Domain scores vary between 4 and 20 (higher scores correspond to better quality of life).

^b^ Statistically significant.

In the univariate analysis, individuals that reported illicit drug use throughout their lives had a higher mean difference in QOL (1.25; SD = 3.19). Individuals with no symptoms of anxiety and depression had a greater mean difference in QOL (2.78; SD = 3.22). Among the laboratory and ART-related variables, no statistically significant association was observed among VL, ARV scheme (TDF/3TC/EFV; TDF/3TC+DTG and other schemes), treatment duration, adherence to treatment, ADR, number of ADR and change in Mean QOL ( Table 3 ).

**Table 3:** Univariate analysis of factors associated with the average mean difference in overall quality of life (QOL) in PLWH in initial ART, Belo Horizonte, state of Minas Gerais (n = 323).

	n	Baseline interview – mean (SD)	Second follow-up interview – mean (SD)	Difference in QOL – mean (SD)	p^b^
Sociodemographic					
Gender					
Male	268	15.69 (2.84)	16.39 (2.82)	0.70 (3.23)	0.054
Female	55	13.85 (3.92)	15.49 (3.34)	1.64 (3.47)	
Age (additional year)	323	-0.01 (0.02)	-0.02 (0.02)	-0.01 (0.02)	0.629
Marital status					
Single, divorced, widow	258	15.23 (3.16)	16.21 (2.81)	0.98 (3.30)	0.177
Married, stable union	65	15.94 (2.89)	16.31 (3.36)	0.37 (3.18)	
Skin color					
White	74	16.14 (2.57)	16.89 (2.75)	0.76 (3.05)	0.807
Non-white	246	15.17 (3.19)	16.03 (2.97)	0.86 (3.30)	
Schooling (years)					
≤ 9	75	14.29 (3.36)	15.15 (3.71)	0.85 (4.04)	0.932
from 10 to 12	125	15.34 (3.01)	16.24 (2.60)	0.90 (2.98)	
≥ 12	122	16.07 (2.91)	16.87 (2.49)	0.80 (3.08)	0.922
Children					
No	216	15.66 (2.99)	16.50 (2.73)	0.84 (3.18)	0.055
Yes	107	14.80 (3.31)	15.70 (3.24)	0.90 (3.49)	
Living with other people					
No	62	15.26 (3.01)	15.39 (3.02)	0.13 (3.76)	0.051
Yes	261	15.40 (3.15)	16.44 (2.87)	1.03 (3.14)	
Employment					
No	131	14.58 (3.41)	15.80 (3.19)	1.22 (3.36)	0.103
Yes	192	15.92 (2.78)	16.53 (2.70)	0.61 (3.21)	
Health insurance					
No	235	15.23(3.08)	16.08 (2.51)	0.84 (3.43)	0.871
Yes	88	15.75 (3.22)	16.66 (2.51)	0.91 (2.86)	
Economic status					
C, D, E	193	15.10(3.19)	15.95 (3.06)	0.85 (3.43)	0.994
A, B	122	15.92 (3.94)	16.77 (2.53)	0.85 (3.03)	
Behavioral					
Religious belief					
No	66	15.52 (3.42)	15.79 (3.24)	0.27 (3.61)	0.096
Yes	255	15.31 (3.04)	16.34 (2.84)	1.03 (3.18)	
Current Smoking (baseline interview)					
No	244	15.79 (2.78)	16.48 (2.65)	0.69 (3.04)	0.098
Yes	79	14.10 (3.73)	15.49 (3.56)	1.39 (3.91)	
Alcohol consumption in the month before the baseline interview					
No	110	15.47 (3.03)	16.16 (3.01)	0.69 (3.34)	0.514
Yes	212	15.33 (3.18)	16.27 (2.90)	0.94 (3.26)	
Illicit drug use in life					
No	169	15.66 (2.95)	16.17 (2.95)	0.51 (3.34)	0.042^a^
Yes	153	15.11 (3.24)	16.37 (2.84)	1.25 (3.19)	
Category of risk/exposure					
Others	114	14.84 (3.42)	15.72 (2.93)	0.88 (3.31)	0.984
MSM	168	15.73 (2.89)	16.60 (2.69)	0.87 (3.27)	
Clinics					
Clinical classification					
Aids	58	15.66 (3.00)	16.28 (3.38)	0.62 (3.84)	0.539
Symptomatic or asymptomatic	260	15.29 (3.11)	16.21 (2.82)	0.92 (3.17)	
resence of signs or symptoms of anxiety or depression during follow-up					
Never	41	13.71 (3.27)	16.49 (2.27)	2.78 (3.22)	
Ever	244	15.55 (3.12)	16.39 (2.88)	0.84 (3.05)	< 0.001^a^
Always	36	16.00 (2.29)	14.78 (3.55)	-1.22 (3.71)	< 0.001^a^
Self-reported comorbidities					
No	264	15.62 (2.99)	16.45 (2.61)	0.83 (3.12)	0.752
Yes	59	14.27 (3.47)	15.25 (3.93)	0.98 (3.97)	
Self-reported co-infections					
No	296	15.46 (3.16)	16.35 (2.84)	0.89 (3.21)	0.284
Yes	25	14.64 (2.50)	14.80 (3.70)	0.16 (3.95)	
HIV diagnosis time (additional month)	322	< 0.01 (0.01)	< 0.01 (0.01)	< -0.01 (0.01)	0.675
Laboratorial					
VL – baseline interview					
Detectable	291	15.43 (3.15)	16.26 (2.93)	0.83 (3.30)	0.064
Undetectable	5	12.80 (2.28)	16.40 (2.19)	3.60 (3.29)	
VL – second follow-up interview^c^					
Detectable	43	14.65 (3.14)	16.14 (2.37)	1.49 (3.03)	0.085
Undetectable	237	15.66 (2.93)	16.20 (3.00)	0.54 (3.35)	
VL (copies/mL)					
Up to 100,000	212	15.31 (3.18)	16.22 (2.88)	0.91 (3.30)	0.823
Higher than 100,000	84	15.57 (3.07)	16.38 (3.02)	0.81 (3.37)	
ART-related					
Antiretroviral scheme					
TDF/3TC/EFV	204	15.27 (3.18)	16.11 (2.82)	0.83 (3.44)	0.635
TDF/3TC + DTG	104	15.75 (2.96)	16.77 (2.59)	1.02 (2.83)	
Other schemes	15	14.13 (3.16)	14.27 (5.06)	0.13 (4.10)	0.453
ART treatment time (additional day)	323	< -0.01 (< 0.01)	< -0.01 (< 0.01)	< -0.01 (< 0.01)	0.306
Adherence – baseline interview					
No	163	15.10 (3.27)	15.73 (3.24)	0.63 (3.43)	0.270
Yes	146	15.71 (2.98)	16.75 (2.50)	1.04 (3.14)	
Adherence – second follow-up interview					
No	191	15.20 (3.38)	15.92 (3.04)	0.71 (3.44)	0.394
Yes	130	15.68 (2.67)	16.71 (2.73)	1.03 (3.04)	
ADR – baseline interview					
No	44	16.46 (3.22)	17.00 (2.42)	0.55 (3.09)	0.503
Yes	267	15.18 (3.12)	16.09 (3.02)	0.91 (3.35)	
ADR – second follow-up interview					
No	155	15.79 (2.63)	16.66 (2.76)	0.86 (3.13)	0.913
Yes	165	15.04 (3.48)	15.87 (3.05)	0.82 (3.44)	
Additional ADR – baseline interview	311	-0.26 (0.06)	-0.16 (0.05)	0.10 (0.97)	0.086
ADR additional – second follow-up interview	323	-0.23 (0.06)	-0.27 (0.06)	-0.04 (0.07)	0.527

QOL: quality of life; PLWH: person living with HIV; SD = standard deviation; LT-CD4 +: T-CD4 + lymphocytes; MSM: men that have sex with men; TDF: tenofovir; 3TC: lamivudine; EFV: efavirenz; DTG: dolutegravir; ART: antiretroviral therapy; ADR: adverse drug reaction; HIV: human immunodeficiency virus; other risks: hemophiliacs, transfusion and occupational.

^a^ Statistically significant.

^b^ The p-values refer to the comparison between the differences in the increase in QOL according to each variable.

^c^ Undetectable VL: < 50 copies/ml.

In the final multiple linear regression model, individuals with religious belief (0.92; 95%CI 0.20 to 1.64) and those that lived with others (1.00; 95%CI 0.26 to 1.65) showed an increase in QOL. Whereas individuals that reported signs or symptoms of anxiety and depression in life (-0.99; 95%CI -1.89 to -0.10) or always (-2.91; 95%CI -4.11 to -1.70) had a reduction in QOL. With each additional ADR reported at baseline, there was a reduction in the individual’s QOL (-0.09; 95%CI -0.18 to 0.01). Those individuals with higher QOL scores in the baseline interview obtained lower QOL increments at the end of the study, thus showing an inversely proportional Association (-0.60; 95%CI -0.69 to -0.50) ( Table 4 ).

**Table 4:** Final multivariate model of factors associated with the difference in overall QOL in PLHIV in initial ART, Belo Horizonte, state of Minas Gerais (n = 307*).

	Coefficient	95%CI		p
Constant	9.887	8.006	11.768	< 0.001
Religious Belief (yes)	0.922	0.199	1.645	0.013
Living with other people (yes)	1.00	0.26	1.65	0.008
Signs or symptoms of anxiety or depression (ever *versus* never)	-0.993	-1.891	-0.096	0.0030
Signs or symptoms of anxiety or depression (always *versus* never)	-2.906	-4.111	-1.701	0.000
Additional adverse reaction reported in baseline interview	-0.088	-0.184	0.007	0.070
Additional score in overall QOL reported in the baseline interview	-0.596	-0.692	-0.500	< 0.001

QOL: quality of life; PLWH: person living with HIV; ART: antiretroviral therapy.

* 16 patients with missing data in covariates were excluded from the final model.

Regarding the adequacy of the multiple linear regression model, the adjusted R^2^ statistic was equal to 0.414, that is, the model explained approximately 41.4% of the variance of the QOL increase. The Durbin-Watson statistic was equal to 2.02, indicating no correlation between the residues. In the collinearity statistics, all predictors had tolerance values above 0.56 and VIF values close to 1 (1.00 to 1.78). The distribution of standardized waste was also verified, and they approached the normal distribution. This assumption was also confirmed by the Q-Q normal residue regression graph. Finally, the dispersion graph between the standardized and predicted residues did not show that they were randomly distributed; therefore, the developed model shows good fit.

## DISCUSSION

Individuals initiating ART in this cohort showed an increase in QOL; having a religious belief and living with other people were positively associated with QOL, whereas having signs or symptoms of anxiety and depression, anytime or always, and additional ADR reported were predictors associated with worse QOL.

The characteristics of this population were similar to those of other studies^11 , 12^ and epidemiological bulletins^13 , 14^ , with a predominance of male, young, self-declared non-white skin color and with high schooling.

Our study showed that, over time, individuals in initial use of ART presented an increase in the overall QOL score, consistent with previous research^15^ . The greatest mean difference in QOL observed in the physical domain, in which aspects such as pain, discomfort, energy, sleep and rest are evaluated, may be related to the use of ART. This causes changes in the course of infection, such as slowing the progression of immunodeficiency caused by the HIV virus and restoring the immune response of individuals – consequently, it can positively affect the individual’s life^18^ .

In the social relations domain, in which questions related to acceptance of diagnosis, family support, interpersonal relationship and sexual life were evaluated, a lower mean difference in QOL was observed. The social relationship is a complication for PLWH, since infection with the virus can be a stigmatizing health condition, which involves feelings of discrimination and, consequently, lack of social support and feelings of loneliness. Considering that these factors will hardly be influenced by the use of ART, it is important to emphasize the need to improve personal relationships and social support for these individuals^15^ .

We observed a statistically significant increase in the overall perception component of QOL. The benefits of ART in the QOL can be explained by the reduction of clinical symptoms of infection. That is, this reinforces the need to link and retain individuals to the health care service and maintain adherence to treatment.

Some behavioral, sociodemographic, clinical and ART-related factors showed a relationship with the change in QOL in individuals initiating ART, and these findings are consistent with the scientific literature^15^ .

Feelings of guilt, loneliness and fear of death, commonly reported by PLWH, are related to spiritual practice and having religious beliefs^12 , 21^ , which contributes to the increase in QOL and health. Faith and religious beliefs are important strategies for coping with health conditions such as HIV, when allied to ART^11 , 21^ .

Our results stresses the importance of family involvement in reducing HIV-related stigma and prejudice. In another study using the same instrument, but with a cross-sectional design^22^ , conducted in the Southern Brazil, individuals that lived alone presented 30% propensity to worse QOL in the social relations domain. Less emotional support during treatment could partially explain the results. Another cross-sectional study, conducted with 100 PLWH in Nepal, using the WHOQOL-bref instrument, demonstrated that good family support has a greater influence on QOL increase, being statistically associated with overall QOL^23^ .

Signs or symptoms of anxiety and depression can impair PLWH QOL^24^ . Depression and anxiety are more frequent in PLWH than in the general population^25^ , which can negatively influence the individual’s behavior and contribute treatment-related difficulties and even worse adherence to ART^26^ . The impact of psychiatric symptoms on PLWH QOL also occurs due to a deterioration of the immune system and consequent increase in disease progression, justified by the increased level of stress and depressive symptoms^27^ . Although HADS is not used for diagnostic purposes, but to evaluate signs and symptoms of anxiety and depression, an association was observed between these signs and symptoms and the reduction of QOL in PLWH in follow-up interviews. These results emphasize the importance of the follow-up by a health professional (psychologist, psychiatrist, among others) in HIV specialized services. In another cross-sectional study on QOL of the Ecoart group, conducted with 366 individuals in Belo Horizonte, being single (unmarried), having other comorbidities, lower educational level, being smoker, and having signs and symptoms of anxiety and depression was associated with lower QOL^4^ . In another cross-sectional study including 150PLWH conducted in Cambodia, the authors suggest a strong association between the increase in QOL assessed by the overall QOL score and the six domains of WHOQOL-HIV bref and the absence of signs or symptoms of anxiety and depression^28^ .

In our study, we verified a greater reduction in QOL to each additional ADR reported by subjects in treatment, that is, the greater the number of ADR reported, the greater the reduction in QOL. This result is similar to that of another study conducted in Brazil, which described the lower number of ADR to ART as one of the factors associated with QOL improvement^29^ . ADR can affect physically and psychologically the performance of professional and personal activities and, consequently, the QOL of individuals under treatment^5^ . Monitoring the use of antiretrovirals is an important tool to assess the safety of these medicines in clinical practice^5^ and ensure better care and satisfaction of individuals with treatment^29^ .

When comparing individuals using the TDF/3TC + DTG regimen with those using TDF/3TC/EFV or other regimens, no statistically significant differences were observed in the mean difference in overall QOL, which can be explained by the fact that part of the Ecoart study population had initiated ART even asymptomatic, due to the change of the HIV treatment guidelines by the Brazilian Ministry of Health. Therefore, these individuals may not have recognized the improvement in QOL with the use of ART, regardless of the regimen used.

Our study presents as limitation the incompleteness of data from the control system of laboratory tests (Siscel), such as TCD4+ lymphocyte and VL count. To minimize this limitation, we considered the data for the three-month period before and after the second follow-up interview, resulting in a low percentage of missing data. As positive points, we cite the longitudinal design, and that data were collected with methodological rigor and in representative sites of PLWH of Belo Horizonte, the high number of the sample and the robustness of the final model obtained by multivariate analysis. Moreover, the WHOQOL-HIV bref is an instrument that evaluates QOL in the two weeks prior to the interview; thus, potential memory biases were minimized.

New studies in PLWH with longer follow-up time, using the WHOQOL-HIV bref, as well as other instruments, are necessary to measure the changes in QOL of these individuals and contribute to the direction of actions and interventions of health professionals that may contribute to increasing QOL in PLWH.

We concluded that, over time, PLWH initiating ART showed improvement in QOL. There is a need to monitor and provide health care support, especially for individuals with signs and symptoms of anxiety and depression and that report adverse reactions at the beginning of treatment.

Our study can contribute to the planning and direction of public actions and policies, as well as to identify modifiable factors that can increase the QOL of these individuals.

## References

[1] 1. World Health Organization. Consolidated guidelines on the use of antiretroviral drugs for treating and preventing HIV infection: recommendations for a public health approach. 2. ed. Genebra: WHO; 2016 [cited 2018 May 20]. Available from: http://www.who.int/hiv/pub/arv/arv-2016/en/. 27466667

[2] 2. Brasil. Ministério da Saúde, Secretaria de Vigilância em Saúde, Departamento de Vigilância, Prevenção e Controle das Infecções Sexualmente Transmissíveis, do HIV/aids e das Hepatites Virais. Protocolo clínico e diretrizes terapêuticas para manejo da infecção pelo HIV em adultos. Brasília; 2017.

[3] 3. Degroote S, Vogelaers D, Vandijck DM. What determines health-related quality of life among people living with HIV: an updated review of the literature. Arch Public Health. 2014;72(1). 10.1186/2049-3258-72-40. PMC4323115 25671112

[4] 4. Costa JO, Pearson SA, Acurcio FA, Bonolo PF, Silveira MR, Ceccato MGB. Health-related quality of life among HIV-infected patients initiating treatment in Brazil in the single-tablet regimen era. AIDS Care. 2019;31(5):572-81. 10.1080/09540121.2019.1576841. 30727749

[5] 5. Mendes JC et al. Adverse reactions associated with first-line regimens in patient initiating antiretroviral therapy. European Journal of Clinical Pharmacology. 2018;74(8):1077-88. 10.1007/s00228-018-2472. 29740676

[6] 6. Zimpel RR, Fleck MP. (2007). Quality of life in HIV-positive Brazilians: application and validation of the WHOQOL-HIV, Brazilian version. AIDS Care. 2007;19(7), 923-30. 10.1080/09540120701213765. 17712697

[7] 7. Botega NJ, Bio MR, Zomignani MA, Garcia Jr. C, Pereira WAB. Transtornos do humor em enfermaria de clínica médica e validação de escala de medida (HADS) de ansiedade e depressão. Rev. Saúde Pública. 1995:29(5):359-63. 10.1590/s0034-89101995000500004 8731275

[8] 8. Oliveira-Filho AD, Morisky DE, Neves SJ, Costa FA, Junior DPL. The 8-item Morisky Medication Adherence Scale: validation of a Brazilian-Portuguese version in hypertensive adults. Research in Social and Administrative Pharmacy. 2014;10(3):554-61. 10.1016/j.sapharm.2013.10.006. 24268603

[9] 9. Associação Brasileira de Empresas de Pesquisa. Critério de classificação econômica. Brasil: Abep; 2008. [cited 2019 Mar 5]. Available from: http://www.abep. org.

[10] 10. Field A. Descobrindo a estatística usando o SPSS-2. Porto Alegre: Bookman; 2009.

[11] 11. Grangeiro A, Escuder MM, Cassanote AJF, Souza RA, Kalichman AO, Veloso V et al. The HIV-Brazil cohort study: design, methods and participant characteristics. PLoS One. 2014;9(5):e95673. 10.1371/journal.pone.0095673. PMC4006775 24789106

[12] 12. Oliveira FBM, Moura MEB, Araújo TME, Andrade EMLR. Qualidade de vida e fatores associados em pessoas vivendo com HIV/aids. Acta paul. enferm. 2015;28(6):510-6. 10.1590/1982-0194201500086.

[13] 13. Brasil. Ministério da Saúde, Secretaria de Vigilância em Saúde. Boletim epidemiológico de aids e DST. Brasília; 2018. [cited 2019 Feb 3]. Available from: http://www.aids.gov.br/pt-br/pub/2018/boletim-epidemiologico-hivaids-2018.

[14] 14. Minas Gerais. Secretaria Estadual de Saúde, Diretoria de Vigilância Epidemiológica, Coordenação de DST, Aids e Hepatites Virais. Boletim epidemiológico mineiro: análise epidemiológica de HIV/aids. Panorama do ano de 2017. Belo Horizonte; 2018. [cited 2019 Feb 5]. Available from: http://www.saude.mg.gov.br/images/noticias_e_eventos/000_2019/jane_fev_mar/BEM%20Mineiro%202018HIV.pdf.

[15] 15. Bakiono F, Guiguimdé PW, Sanou M, Ouédraogo L, Robert A. Quality of life in persons living with HIV in Burkina Faso: a follow-up over 12 months. BMC Public Health. 2015; 15(1119). 10.1186/s12889-015-2444-4. PMC4643494 26563970

[16] 16. Liu C, Weber K, Robison E, Hu Z, Jacobson LP, Gange SJ. Assessing the effect of HAART on change in quality of life among HIV-infected women. AIDS Research and Therapy. 2006;3(6):1-11. 10.1186/1742-6405-3-6. PMC1459186 16549012

[17] 17. Solomon S, Batavia A, Venkatesh KK, Brown L, Verma P, Cecelia AJ, Daly C, Mahendra VS, Kumarasamy N, Mayer KH. A longitudinal quality-of-life study of HIV-infected persons in South India: the case for comprehensive clinical care and support services. AIDS Education and Prevention. 2009;21(2):104-12. 10.1521/aeap.2009.21.2.104. 19397433

[18] 18. Greco DB, Pedroso ERP, Westin MR. Síndrome da imunodeficiência adquirida. In: Pedroso ERP. Série medicina interna: doenças infecciosas. Rio de Janeiro: Rubio; 2015. p. 324-73.

[19] 19. Dutra BS, Lédo AP, Lins-Kusterer L, Luz E, Prieto IR, Brites C. Changes health-related quality of life in HIV-infected patients following initiation of antiretroviral therapy: a longitudinal study. Braz J Infect Dis. 2019;23(4):211-7. 10.1016/j.bjid.2019.06.005. PMC9428026 31344351

[20] 20. Gruszczyńska E, Rzeszutek M. Trajectories of health-related quality of life and perceived social support among people living with HIV undergoing antiretroviral treatment: does gender matter?. Front Psychol. 2019;23(10):1664. 10.3389/fpsyg.2019.01664. PMC6664262 31396129

[21] 21. Medeiros B, Saldanha AAW. Religiosidade e qualidade de vida em pessoas com HIV. Estud. psicol. 2012;29(1):53-61. 10.1590/S0103-166X2012000100006.

[22] 22. Razera F, Ferreira J, Bonamigo RR. Factors associated with health-related quality-of-life in HIV-infected Brazilians. International Journal of STD & AIDS. 2008;19(8):519-23. 10.1258/ijsa.2008.007289. 18663036

[23] 23. Baral R, Thapa U, Khatiwada D. Quality of life among people living with human immunodeficiency virus and acquired immune deficiency syndrome in an anti-retroviral therapy clinic. J Nepal Health Res Counc. 2019;16(41):405-9. 30739930

[24] 24. Tostes MA, Chalub M, Botega NJ. The quality of life of HIV-infected women is associated with psychiatric morbidity. AIDS Care. 2004;16(2):177-86. 10.1080/09540120410001641020. 14676024

[25] 25. Souza Junior PRB, Szwarcwald CL, Castilho EA. Self-rated health by HIV-infected individuals undergoing antiretroviral therapy in Brazil. Cad. Saúde Pública. 2011;27(Suppl 1):s56-s66. 10.1590/S0102-311X2011001300007. 21503525

[26] 26. Reis AC, Lencastre L, Guerra MP, Remor E. Relação entre sintomatologia psicopatológica, adesão ao tratamento e qualidade de vida na infecção HIV e AIDS. Psicol. Reflex. Crit. 2010;23(3):420-9. 10.1590/S0102-79722010000300002.

[27] 27. Leserman J. Role of depression, stress, and trauma in HIV disease progression. Psychosom Med. 2008;70(5):539-45. 10.1097/PSY.0b013e3181777a5f. 18519880

[28] 28. Yang Y, Thai S, Choi J. An evaluation of quality of life among Cambodian adults living with HIV/AIDS and using antiretroviral therapy: a short report. AIDS Care. 2016;28(12):1546-50. 10.1080/09540121.2016.1192100. 27285879

[29] 29. Campos LN, Cesar CC, Guimarães MD. Quality of life among HIV-infected patients in Brazil after initiation of treatment. Clinics. 2009;64(9):867-75. 10.1590/S1807-59322009000900007. PMC2745298 19759880

