# Supplementary material for: Quality of life in individuals initiating antiretroviral therapy: a cohort study
Source: Rev Saude Publica. 2020 Dec 4;54:146. doi: 10.11606/s1518-8787.2020054001920 (PMC7726918; doi:10.11606/s1518-8787.2020054001920)
Supplement: Translation

# Translation: Qualidade de vida em indivíduos iniciando a terapia antirretroviral: um estudo de coorte

**Authors:** Gabriela Sales Pimentel, Maria das Graças Braga Ceccato, Juliana de Oliveira Costa, Jullye Campos Mendes, Palmira de Fátima Bonolo, Micheline Rosa Silveira

**Affiliations:** I Brasil Universidade Federal de Minas Gerais. Faculdade de Farmácia. Programa de Pós-Graduação em Medicamentos e Assistência Farmacêutica. Belo Horizonte, MG, Brasil; II Brasil Universidade Federal de Minas Gerais. Faculdade de Farmácia. Departamento de Farmácia Social. Belo Horizonte, MG, Brasil; III Australia University of New South Wales. Centre for Big Data Research in Health. Faculty of Medicine. Sydney, Australia; IV Brasil Universidade Federal de Minas Gerais. Faculdade de Medicina. Pós-Graduação em Saúde Pública. Belo Horizonte, MG, Brasil; V Brasil Universidade Federal de Minas Gerais. Faculdade de Medicina. Departamento de Medicina Preventiva e Social. Belo Horizonte, MG, Brasil

**Keywords:** Fármacos Anti-HIV, Terapia Antirretroviral de Alta Atividade, Qualidade de vida, WHOQOLHIV-bref, Estudos de Coortes

## Abstract

**OBJETIVO:**

Avaliar longitudinalmente a alteração da qualidade de vida em pessoas que vivem com HIV iniciando a terapia antirretroviral, atendidas em três serviços públicos de referência na assistência especializada ao HIV em Belo Horizonte.

**MÉTODOS:**

Estudo de coorte prospectivo, com o acompanhamento de pessoas que vivem com HIV, com 18 anos de idade ou mais, e iniciando terapia antirretroviral. Dados sociodemográficos, comportamentais, clínicos, relacionados ao tratamento farmacológico e ao serviço foram obtidos por entrevistas, complementados com informações dos prontuários clínicos e dos sistemas de informação do Programa Brasileiro de HIV/AIDS. A qualidade de vida foi avaliada utilizando o instrumento WHOQOLHIV-bref, por meio de entrevista face a face, com intervalo mínimo de seis meses entre as entrevistas. A alteração média na qualidade de vida entre as duas entrevistas foi avaliada utilizando o teste *t* pareado. Os fatores associados foram avaliados por meio de regressão linear múltipla.

**RESULTADOS:**

A qualidade de vida global, assim como a qualidade de vida nos domínios físico, psicológico, nível de independência, ambiente e espiritual foram estatisticamente melhores em pessoas que vivem com HIV usando terapia antirretroviral no final do tempo de acompanhamento. Fatores independentemente associados ao incremento na qualidade de vida foram possuir crença religiosa e morar com outras pessoas. Enquanto ter sinais ou sintomas de ansiedade e depressão e o número de reações adversas a medicamentos reportadas foram preditores associados à piora da qualidade de vida.

**CONCLUSÕES:**

Os resultados evidenciam melhora na qualidade de vida em pessoas vivendo com HIV iniciando a terapia antirretroviral ao longo do tempo. Evidenciam ainda a necessidade de se acompanhar e prover cuidados de saúde, em especial para indivíduos com sinais e sintomas de ansiedade e depressão e que relatam reações adversas a medicamentos no início do tratamento.

## INTRODUÇÃO

No Brasil, nos últimos anos, os esquemas antirretrovirais (ARV) de primeira linha foram modificados, seguindo a tendência mundial e recomendações da Organização Mundial da Saúde (OMS)^1^ . Em 2015, o Protocolo Clínico e Diretrizes Terapêuticas (PCDT) para o manejo do HIV passou a recomendar como esquema de primeira linha um medicamento em dose fixa combinada (DFC) contendo os fármacos tenofovir (TDF), lamivudina (3TC) e efavirenz (EFV). Em 2017, o dolutegravir (DTG), fármaco inibidor da integrase, foi inserido no esquema de primeira linha, em substituição ao EFV, juntamente com um comprimido da associação de TDF e 3TC^2^ .

Em países em desenvolvimento, como o Brasil, existem evidências limitadas, em estudos longitudinais, sobre os fatores determinantes da qualidade de vida (QV) em pessoas que vivem com HIV (PVHIV). Em uma revisão de literatura, os fatores sexo, idade, situação familiar, escolaridade, emprego, renda, carga viral, contagem de linfócitos TCD4+, tempo de diagnóstico, presença de sintomas de depressão e ansiedade, apoio social, atenção à saúde, uso de drogas lícitas e ilícitas, adesão à terapia antirretroviral (TARV), estilo de vida e comportamento sexual foram diretamente associados à QV de PVHIV^3^ . Portanto, a compreensão da QV é essencial para analisar o impacto físico e biopsicossocial que o HIV pode ocasionar nos indivíduos, possibilitando maior conhecimento acerca de si mesmo, de sua adaptação à condição de viver com HIV e de seu tratamento. Considera-se ainda que existem evidências limitadas sobre o impacto do início da TARV na QV em PVHIV.

O objetivo deste estudo foi avaliar longitudinalmente a QV em PVHIV iniciando a TARV atendidas em três serviços públicos de referência em Belo Horizonte, bem como avaliar os preditores de alteração na QV.

## MÉTODOS

Estudo de coorte, no qual foram utilizados dados do projeto Ecoart, cujos detalhes foram previamente publicados^4 , 5^ .

A seleção da amostra foi não aleatória, e todos os indivíduos elegíveis e presentes em três serviços públicos de assistência especializada em HIV, que dispensam TARV para aproximadamente 80% das PVHIV no município de Belo Horizonte, foram convidados a participar do estudo. O recrutamento ocorreu entre setembro de 2015 e outubro de 2017.

Foram incluídos indivíduos com até 180 dias de TARV, em acompanhamento em um dos serviços avaliados e que foram identificados por meio do registro no Sistema de Controle Logístico de Medicamentos (Siclom). Os critérios de inclusão foram assinatura do termo de consentimento livre e esclarecido (TCLE), ter 18 anos ou mais, autonomia para responder à entrevista e ter realizado a entrevista basal e a segunda entrevista de acompanhamento.

Os dados foram obtidos em entrevistas face a face e em bases de dados secundárias, utilizando-se formulários padronizados que continham os instrumentos *World Health Organization Quality of Life HIV –* em sua versão resumida (WHOQOLHIV- *bref* ) –, Escala Hospitalar de Ansiedade e Depressão (HADS) e Escala de Adesão Terapêutica de Morisky de Oito Itens (MMAS-8), todos validados no Brasil^6^ , além de perguntas relativas a informações sociodemográficas, comportamentais, clínicas, laboratoriais, relacionadas ao tratamento farmacológico e ao serviço de saúde.

Para minimizar potenciais riscos de vieses, os pesquisadores foram devidamente treinados e foi realizado projeto piloto antes de iniciar a coleta de dados.

### Variável Dependente

A QV foi avaliada por meio do instrumento WHOQOLHIV- *bref* , em dois momentos, na entrevista basal e na segunda entrevista de acompanhamento, prevista para ocorrer seis meses após a entrevista basal. A diferença média da QV entre esses dois momentos constituiu a variável dependente deste estudo, que apresenta os resultados unicamente daqueles indivíduos que terminaram o acompanhamento. Em um artigo prévio^4^ foram publicados os dados relativos a QV na entrevista basal.

O WHOQOLHIV- *bref* é um instrumento específico para avaliação da QV em PVHIV, que, por meio de 31 perguntas, avalia os domínios: físico, psicológico, nível de independência, relações sociais, meio ambiente e espiritualidade. Cada pergunta possui opções de resposta em escala Likert de cinco pontos, onde 1 indica percepções negativas e 5, percepções positivas, exceto para sete itens em que a escala é invertida. A média dos itens dentro de cada domínio é usada para calcular a pontuação total do domínio. O resultado é multiplicado por 4, e, assim, os escores variam entre 4 e 20^6^ .

O componente QV global e percepção geral da saúde foi construído utilizando as duas primeiras perguntas do instrumento WHOQOLHIV- *bref* . Fez-se a média dos itens, e o resultado foi multiplicado por 4; dessa forma, os escores também variaram de 4 a 20^6^ .

### Variáveis Independentes

Para caracterizar a população do estudo, foram analisadas variáveis sociodemográficas, comportamentais, clínicas e laboratoriais, relacionadas ao tratamento farmacológico e ao serviço.

A presença de sinais ou sintomas de ansiedade e depressão foi mensurada por meio da HADS, composta por 14 itens^7^ . Pessoas que tiveram 8 ou mais pontos em cada um dos agravos foram classificadas com sintomas de ansiedade e depressão. Os indivíduos foram classificados segundo a presença de sinais e sintomas de ansiedade ou depressão na entrevista basal e na entrevista de acompanhamento, sendo categorizados em: (i) nunca apresentaram sinais e sintomas de ansiedade ou depressão, (ii) apresentaram esses sintomas em alguma das duas entrevistas ou (iii) apresentaram sintomas em ambas as entrevistas.

A MMAS-8 foi utilizada para avaliar a adesão a TARV. O escore total da MMAS-8 varia de 0 a 8, sendo que, quanto maior for a pontuação, maior é a adesão^8^ . Foram considerados aderentes os indivíduos que obtiveram escore de 8 pontos. As reações adversas a medicamentos (RAM) foram avaliadas por meio de autorrelato. Foram considerados indivíduos com carga viral (CV) indetectável aqueles que apresentaram valor inferior a 50 cópias por ml.

A variável classe econômica ( *status* socioeconômico) foi avaliada de acordo com critérios brasileiros, como alta (A, B), intermediária (C) e baixa (D e E), na qual os indivíduos são classificados por meio de grupos socioeconômicos por posse de itens de conforto e nível de escolaridade do chefe familiar^9^ .

Para a variável comorbidade, foi considerado o autorrelato de qualquer doença diagnosticada por médico, como hipertensão arterial, diabetes, hipercolesterolemia, entre outras.

### Análise Estatística

A análise descritiva foi realizada por distribuição de frequências para variáveis categóricas e medidas de tendência central para as variáveis quantitativas.

Para avaliar os escores do instrumento WHOQOLHIV- *bref* , foram apresentadas médias e desvio-padrão para cada domínio, relativos à entrevista basal e à segunda entrevista de acompanhamento. O teste *t* pareado foi utilizado para comparar as diferenças médias dos escores de QV entre as duas entrevistas.

A análise univariável da relação entre variáveis independentes e a alteração no escore de QV foi avaliada pelo teste *t* Student (comparação de médias) e o teste Mann-Whitney (comparação de medianas).

A associação foi analisada pelo ajuste de um modelo de regressão linear múltipla. Foram selecionadas para entrar no modelo multivariável as variáveis que apresentaram valor *p* igual ou inferior a 0,20.

O método *backward stepwise* foi utilizado para obtenção do modelo final. Os resultados da regressão linear múltipla foram demonstrados por meio de coeficientes da regressão, com seus respectivos intervalos de confiança a 95% (IC95%).

A adequação do modelo foi avaliada por um conjunto de estatísticas. As estatísticas R^2^e R^2^ajustado foram utilizadas para verificar a porcentagem da variância relacionada ao incremento na QV explicada pelo modelo. A estatística de Durbin- Watson foi utilizada para verificar o pressuposto de que os resíduos não estão correlacionados, com valores entre 0 e 4, sendo que 2 significa ausência de correlação entre os resíduos. Foi testado também se havia multicolinearidade no modelo final, utilizando as estatísticas de tolerância (aceitável > 0,10) e VIF (aceitável < 10)^10^ . Para avaliar se os resíduos tinham distribuição normal, foram realizados os seguintes gráficos: resíduos padronizados da regressão por valores previstos da regressão padronizados, histograma de frequências dos resíduos padronizados da regressão, e um gráfico percentil-percentil (P.P. plot).

Foi utilizado o *software* Statistical Package for the Social Sciences (SPSS) versão 22.0. Em todas as análises considerou-se o nível de significância de 0,05.

### Aspectos Éticos

O projeto de pesquisa Ecoart foi aprovado pelo Comitê de Ética em Pesquisa (COEP) da Universidade Federal de Minas Gerais (protocolo CAAE 31192914.3.3001.5124, parecer CEP 769.085) e dos serviços participantes. A pesquisa foi realizada seguindo instruções da Resolução 466/2012 do Conselho Nacional de Saúde.

## RESULTADOS

Durante todo o período de recrutamento do projeto Ecoart, foram identificados 1.017 indivíduos atendidos nos três serviços. Desses, 468 realizaram a entrevista basal, e 323 (69,0%) realizaram a segunda entrevista de acompanhamento e foram incluídos nesse estudo ( Figura 1 ).

**Figura:** Diagrama de inclusão dos indivíduos no estudo.

Ao comparar o grupo que completou a segunda entrevista de acompanhamento com aqueles que não a realizaram, não houve diferenças estatisticamente significantes em relação a sexo, idade, estado civil e escolaridade. Também não houve diferenças entre os indivíduos na entrevista basal quanto à QV geral e nos domínios de QV.

A maioria dos entrevistados era do sexo masculino (83,0%), sem cônjuge (79,9%), sem filhos (66,9%), entre 20 e 34 anos (54,5%), não possuía plano privado de saúde (72,8%) e se autodeclarou como não branco (76,9%). Verificou-se que 80,8% dos indivíduos residiam com outras pessoas, 38,8% possuíam de 10 a 12 anos de escolaridade e 59,4% estavam empregados no momento da entrevista, havendo predomínio da classe econômica C (45,7%). Em relação às características comportamentais e hábitos de vida, 24,5% relataram uso atual de tabaco, 65,8%, uso de álcool recente, e 47,5% declararam já ter utilizado ou utilizar drogas ilícitas na vida. A maioria declarou ter alguma crença religiosa (79,4%). Verificou-se que 59,6% eram homens que fazem sexo com outros homens (HSH). Quanto às características clínicas, 7,8% apresentaram coinfecção (como tuberculose, sífilis, toxoplasmose e candidíase), 18,3^%, uma ou mais comorbidades autorrelatadas, e 35,9%, sinais ou sintomas de ansiedade e depre^ssão. A maioria dos indivíduos apresentou classificação clínica assintomática (67,3%) e tinha tempo de diagnóstico de infecção pelo HIV menor ou igual a seis meses (61,8 %). No que diz respeito às características laboratoriais basais, 23,5% apresentaram contagem inicial de linfócitos TCD4^+^inferior a 200 células/mm^3^, 90,1%, CV detectável, e 26,0%, CV superior a 100.000 cópias/ml. Em relação ao tratamento farmacológico, 63,2% utilizaram TDF/3TC/EFV, e 32,2%, TDF/3TC + DTG. A maioria (52,3%) estava em uso de TARV há 60 dias ou menos, eram não aderentes a TARV (52,8%), apresentaram RAM (85,9%), com três ou menos RAM (53,1%) ( Tabela 1 ).

**Tabela 1:** Características sociodemográficas, comportamentais, clínicas, laboratoriais, terapêuticas e relacionadas ao serviço de PVHIV em TARV inicial na entrevista basal e na segunda entrevista de acompanhamento, Belo Horizonte, Minas Gerais (n = 323).

Entrevista basal		
Características	n	(%)
Sociodemográficas		
Sexo (masculino)	268	83
Idade (anos)		
18–19	10	3,1
20–34	176	54,5
35–49	102	31,6
≥ 50	35	10,8
Idade (média (DP)), anos	34,5 (10,7)	
Estado civil (solteiro/divorciado/viúvo)	258	79,9
Cor de pele (não branco)	246	76,9
Escolaridade (anos)		
≤ 9	75	23,3
10–12	125	38,8
≥ 13	122	37,9
Filhos (não)	216	66,9
Reside com outras pessoas (sim)	261	80,8
Emprego (sim)	192	59,4
Plano de saúde (não)	235	72,8
Classe econômica		
A, B	122	38,7
C	144	45,7
D, E	49	15,6
Comportamentais		
Crença religiosa (sim)	255	79,4
Tabagismo atual (sim)	79	24,5
Consumo de álcool no último mês (sim)	212	65,8
Uso de droga ilícita na vida (sim)	153	47,5
Categoria de risco/exposição (HSH)	168	59,6
Clínicas		
Classificação clínica (assintomática)	214	67,3
Sinais ou sintomas de ansiedade ou depressão (sim)	116	35,9
Comorbidades autorrelatadas (não)	264	81,7
Coinfecções autorrelatadas (não)	296	92,2
Tempo de diagnóstico de HIV (média (DP)), meses	77,1 (59,8)	
Laboratoriais		
Contagem de linfócitos TCD4+ (células/mm3)		
< 200	76	23,5
200–500	124	38,4
> 500	96	29,7
Dados faltantes	27	8,4
CV*		
Detectável	291	90,1
Indetectável	5	1,5
Dados faltantes	27	8,4
CV (cópias/mL)		
≤ 100.000	212	65,6
> 100.000	84	26
Dados faltantes	27	8,4
Relacionadas à TARV		
Esquema antirretroviral		
TDF/3TC/EFV	204	63,2
TDF/3TC + DTG	104	32,2
Outros esquemas	15	4,6
Tempo de tratamento ART (média (DP)), dias	77,1 (59,8)	
Adesão (não)	163	52,8
RAM (sim)	267	85,9
Número de RAM (média (DP))	3,8 (3,2)	
Relacionadas ao serviço		
Serviço de saúde de acompanhamento		
I	106	32,8
II	144	44,6
III	73	22,6
Segunda entrevista de acompanhamento		
Clínicas		
Sinais ou sintomas de ansiedade ou depressão		
Nunca (nem na basal, nem na entrevista de acompanhamento)	41	12,7
Alguma vez (basal ou entrevista de acompanhamento)	244	75,5
Sempre (basal e entrevista de acompanhamento)	36	11.1
Laboratoriais		
CV (supressão viral)		
Detectável	43	13,3
Indetectável	237	73,4
Dados faltantes	43	13,3
Relacionadas à TARV		
Adesão (não)	191	59,5
RAM (sim)	165	51,6
Número de RAM (média (DP))	1,9 (2,7)	

LT-CD4+: Linfócitos T-CD4+; HSH: homens que fazem sexo com homens; TDF: tenofovir; 3TC: lamivudina; EFV: efavirenz; DTG: dolutegravir; TARV: terapia antirretroviral; RAM: reação adversa a medicamento; HIV: human immunodeficiency virus; Outros riscos: hemofílicos, transfusão e ocupacional.

* CV indetectável: < 50 cópias/ml.

Na segunda entrevista de acompanhamento, em relação às características clínicas, laboratoriais e relacionadas a TARV, 34,3% dos indivíduos apresentaram sinais e sintomas de ansiedade e depressão, 73,4%, CV indetectável (supressão viral), 59,5% relataram não adesão tratamento, e 51,6% relataram alguma RAM, sendo que 79,9% relataram três ou menos RAM ( Tabela 1 ).

Os dados de QV foram obtidos na entrevista basal e na segunda entrevista de acompanhamento, que ocorreu em média 9,47 meses (mediana = 8,66; mínimo = 3,5; máximo = 23,0; intervalo interquartil = 19,5, DP = 3,67) após a entrevista basal.

Foram observadas, ao longo do tempo, melhorias estatisticamente significantes na QV global e nos domínios, físico, psicológico, nível de independência, ambiente e espiritual em indivíduos iniciando TARV. O único domínio em que a diferença média entre as entrevistas basal e de acompanhamento não foi significante foi o das relações sociais.

Os domínios de QV com maiores diferenças médias foram o físico (5,11; DP = -3,75), espiritual (3,23; DP = 6,20) e psicológico (1,32; DP = 2,82) ( Tabela 2 ).

**Tabela 2:** Distribuição dos escores dos domínios do WHOQOLHIV- *bref* na entrevista basal e após o acompanhamento em indivíduos vivendo com HIV em TARV inicial, Belo Horizonte, Minas Gerais (n = 323).

Domínios WHOQOLHIV-bref^a^	n	Entrevista basal – média (DP)	Segunda entrevista de acompanhamento – média (DP)	Diferença da média da segunda entrevista e da entrevista basal (DP)	p
Qualidade de vida global	323	15,37 (3,12)	16,24 (2,93)	0,86 (3,28)	< 0,001^b^
Físico	314	10,94 (2,20)	16,04 (2,94)	5,11 (3,75)	< 0,001^b^
Psicológico	318	14,03 (2,06)	15,35 (2,73)	1,32 (2,82)	< 0,001^b^
Nível de independência	316	14,89 (2,26)	15,72 (2,86)	0,82 (3,08)	< 0,001^b^
Relações sociais	295	15,28 (3,01)	15,57 (2,83)	0,29 (3,15)	0,113
Ambiente	315	14,29 (2,40)	14,71 (2,36)	0,42 (2,08)	< 0,001^b^
Espiritual	315	11,67 (3,42)	14,89 (3,73)	3,23 (6,20)	< 0,001^b^

DP: desvio-padrão; TARV: terapia antirretroviral.

^a^Escores dos domínios variam de 4 a 20 (maiores escores correspondem a melhor qualidade de vida).

^b^Estatisticamente significante.

Na análise univariável, indivíduos que relataram uso de drogas ilícitas durante toda a vida apresentaram diferença média na QV mais elevada (1,25; DP = 3,19). Indivíduos com ausência de sintomas de ansiedade e depressão apresentaram maior diferença média na QV (2,78; DP = 3,22). Dentre as variáveis laboratoriais e relacionadas a TARV, não foi observada associação estatisticamente significativa entre CV, esquema ARV (TDF/3TC/EFV; TDF/3TC + DTG e outros esquemas), tempo de tratamento, adesão ao tratamento, RAM, número de RAM e alteração da QV média ( Tabela 3 ).

**Tabela 3:** Análise univariável de fatores associados com a diferença média da qualidade de vida (QV) geral em PVHIV em TARV inicial, Belo Horizonte, Minas Gerais (n = 323).

	n	Entrevista basal – média (DP)	Segunda entrevista de acompanhamento – média (DP)	Diferença na QV – média (DP)	p^b^
Sociodemográficas					
Sexo					
Masculino	268	15,69 (2,84)	16,39 (2,82)	0,70 (3,23)	0,054
Feminino	55	13,85 (3,92)	15,49 (3,34)	1,64 (3,47)	
Idade (ano adicional)	323	-0,01 (0,02)	-0,02 (0,02)	-0,01 (0,02)	0,629
Estado civil					
Solteiro, divorciado, viúvo	258	15,23 (3,16)	16,21 (2,81)	0,98 (3,30)	0,177
Casado, união estável	65	15,94 (2,89)	16,31 (3,36)	0,37 (3,18)	
Cor de pele					
Branco	74	16,14 (2,57)	16,89 (2,75)	0,76 (3,05)	0,807
Não branco	246	15,17 (3,19)	16,03 (2,97)	0,86 (3,30)	
Escolaridade (anos)					
≤ 9	75	14,29 (3,36)	15,15 (3,71)	0,85 (4,04)	0,932
de 10 a 12	125	15,34 (3,01)	16,24 (2,60)	0,90 (2,98)	
≥ 12	122	16,07 (2,91)	16,87 (2,49)	0,80 (3,08)	0,922
Filhos					
Não	216	15,66 (2,99)	16,50 (2,73)	0,84 (3,18)	0,055
Sim	107	14,80 (3,31)	15,70 (3,24)	0,90 (3,49)	
Reside com outras pessoas					
Não	62	15,26 (3,01)	15,39 (3,02)	0,13 (3,76)	0,051
Sim	261	15,40 (3,15)	16,44 (2,87)	1,03 (3,14)	
Emprego					
Não	131	14,58 (3,41)	15,80 (3,19)	1,22 (3,36)	0,103
Sim	192	15,92 (2,78)	16,53 (2,70)	0,61 (3,21)	
Plano de saúde					
Não	235	15,23(3,08)	16,08 (2,51)	0,84 (3,43)	0,871
Sim	88	15,75 (3,22)	16,66 (2,51)	0,91 (2,86)	
Classe econômica					
C, D, E	193	15,10(3,19)	15,95 (3,06)	0,85 (3,43)	0,994
A, B	122	15,92 (3,94)	16,77 (2,53)	0,85 (3,03)	
Comportamentais					
Crença religiosa					
Não	66	15,52 (3,42)	15,79 (3,24)	0,27 (3,61)	0,096
Sim	255	15,31 (3,04)	16,34 (2,84)	1,03 (3,18)	
Tabagismo atual (entrevista basal)					
Não	244	15,79 (2,78)	16,48 (2,65)	0,69 (3,04)	0,098
Sim	79	14,10 (3,73)	15,49 (3,56)	1,39 (3,91)	
Consumo de álcool no mês anterior à entrevista basal					
Não	110	15,47 (3,03)	16,16 (3,01)	0,69 (3,34)	0,514
Sim	212	15,33 (3,18)	16,27 (2,90)	0,94 (3,26)	
Uso de droga ilícita na vida					
Não	169	15,66 (2,95)	16,17 (2,95)	0,51 (3,34)	0,042^a^
Sim	153	15,11 (3,24)	16,37 (2,84)	1,25 (3,19)	
Categoria de risco/exposição					
Outros	114	14,84 (3,42)	15,72 (2,93)	0,88 (3,31)	0,984
HSH	168	15,73 (2,89)	16,60 (2,69)	0,87 (3,27)	
Clínicas					
Classificação clínica					
Aids	58	15,66 (3,00)	16,28 (3,38)	0,62 (3,84)	0,539
Sintomático ou assintomático	260	15,29 (3,11)	16,21 (2,82)	0,92 (3,17)	
Presença de sinais ou sintomas de ansiedade ou depressão durante o acompanhamento					
Nunca	41	13,71 (3,27)	16,49 (2,27)	2,78 (3,22)	
Alguma vez	244	15,55 (3,12)	16,39 (2,88)	0,84 (3,05)	< 0,001^a^
Sempre	36	16,00 (2,29)	14,78 (3,55)	-1,22 (3,71)	< 0,001^a^
Comorbidades autorrelatadas					
Não	264	15,62 (2,99)	16,45 (2,61)	0,83 (3,12)	0,752
Sim	59	14,27 (3,47)	15,25 (3,93)	0,98 (3,97)	
Coinfecções autorrelatadas					
Não	296	15,46 (3,16)	16,35 (2,84)	0,89 (3,21)	0,284
Sim	25	14,64 (2,50)	14,80 (3,70)	0,16 (3,95)	
Tempo de diagnóstico do HIV (mês adicional)	322	< 0,01 (0,01)	< 0,01 (0,01)	< -0,01 (0,01)	0,675
Laboratoriais					
CV – entrevista basal					
Detectável	291	15,43 (3,15)	16,26 (2,93)	0,83 (3,30)	0,064
Indetectável	5	12,80 (2,28)	16,40 (2,19)	3,60 (3,29)	
CV – segunda entrevista de acompanhamento^c^					
Detectável	43	14,65 (3,14)	16,14 (2,37)	1,49 (3,03)	0,085
Indetectável	237	15,66 (2,93)	16,20 (3,00)	0,54 (3,35)	
CV (cópias/mL)					
Até 100 mil	212	15,31 (3,18)	16,22 (2,88)	0,91 (3,30)	0,823
Acima de 100 mil	84	15,57 (3,07)	16,38 (3,02)	0,81 (3,37)	
Relacionadas à TARV					
Esquema antirretroviral					
TDF/3TC/EFV	204	15,27 (3,18)	16,11 (2,82)	0,83 (3,44)	0,635
TDF/3TC + DTG	104	15,75 (2,96)	16,77 (2,59)	1,02 (2,83)	
Outros esquemas	15	14,13 (3,16)	14,27 (5,06)	0,13 (4,10)	0,453
Tempo de tratamento ART (dia adicional)	323	< -0,01 (< 0,01)	< -0,01 (< 0,01)	< -0,01 (< 0,01)	0,306
Adesão – entrevista basal					
Não	163	15,10 (3,27)	15,73 (3,24)	0,63 (3,43)	0,270
Sim	146	15,71 (2,98)	16,75 (2,50)	1,04 (3,14)	
Adesão – segunda entrevista de acompanhamento					
Não	191	15,20 (3,38)	15,92 (3,04)	0,71 (3,44)	0,394
Sim	130	15,68 (2,67)	16,71 (2,73)	1,03 (3,04)	
RAM – entrevista basal					
Não	44	16,46 (3,22)	17,00 (2,42)	0,55 (3,09)	0,503
Sim	267	15,18 (3,12)	16,09 (3,02)	0,91 (3,35)	
RAM – segunda entrevista de acompanhamento					
Não	155	15,79 (2,63)	16,66 (2,76)	0,86 (3,13)	0,913
Sim	165	15,04 (3,48)	15,87 (3,05)	0,82 (3,44)	
RAM adicional – Entrevista basal	311	-0,26 (0,06)	-0,16 (0,05)	0,10 (0,97)	0,086
RAM adicional – Segunda entrevista de acompanhamento	323	-0,23 (0,06)	-0,27 (0,06)	-0,04 (0,07)	0,527

QV: qualidade de vida; PVHIV: pessoa vivendo com HIV; DP = desvio-padrão; LT-CD4+: linfócitos T-CD4+; HSH: homens que fazem sexo com homens; TDF: tenofovir; 3TC: lamivudina; EFV: efavirenz; DTG: dolutegravir; TARV: terapia antirretroviral; RAM: reação adversa a medicamento; HIV: human immunodeficiency virus; Outros riscos: hemofílicos, transfusão e ocupacional.

^a^Estatisticamente significante.

^b^Os valores de *p* se referem à comparação entre as diferenças no incremento de QV de acordo com cada variável.

^c^CV indetectável: < 50 cópias/ml.

No modelo final de regressão linear múltipla, apresentaram aumento na QV indivíduos que possuíam alguma crença religiosa (0,92; IC95% 0,20 a 1,64) e os que residiam com outras pessoas (1,00; IC95% 0,26 a 1,65). Enquanto indivíduos que reportaram sinais ou sintomas de ansiedade e depressão alguma vez (-0,99; IC95% -1,89 a -0,10) ou sempre (-2,91; IC95% -4,11 a -1,70) apresentaram redução na QV. A cada RAM adicional relatada na entrevista basal houve redução na QV do indivíduo (-0,09; IC95% -0,18 a 0,01). Aqueles indivíduos que apresentaram maiores escores de QV na entrevista basal obtiveram menores incrementos de QV ao final do estudo, mostrando assim uma associação inversamente proporcional (-0,60; IC95% -0,69 a -0,50) ( Tabela 4 ).

**Tabela 4:** Modelo multivariável final dos fatores associados com a diferença na QV geral em PVHIV em TARV inicial, Belo Horizonte, Minas Gerais (n = 307*).

	Coeficiente	IC95%		p
Constante	9,887	8,006	11,768	< 0,001
Crença (sim)	0,922	0,199	1,645	0,013
Reside com outras pessoas (sim)	1,00	0,26	1,65	0,008
Sinais ou sintomas de ansiedade ou depressão (alguma vez *versus* nunca)	-0,993	-1,891	-0,096	0,0030
Sinais ou sintomas de ansiedade ou depressão (sempre *versus* nunca)	-2,906	-4,111	-1,701	0,000
Reação adversa adicional reportada na entrevista basal	-0,088	-0,184	0,007	0,070
Escore adicional na QV Global reportada na entrevista basal	-0,596	-0,692	-0,500	< 0,001

QV: qualidade de vida; PVHIV: pessoa vivendo com HIV; TARV: terapia antirretroviral.

* 16 pacientes com dados faltantes em covariáveis foram excluídos do modelo final.

Com relação à adequação do modelo de regressão linear múltipla, a estatística de R^2^ajustado foi igual a 0,414, ou seja, o modelo explicou aproximadamente 41% da variância do incremento da QV. A estatística de Durbin-Watson foi igual a 2,02, indicando que não houve correlação entre os resíduos. Nas estatísticas de colinearidade, todos os preditores tiveram valores de tolerância acima de 0,56 e valores de VIF próximos de 1 (1,00 a 1,78). Foi verificada também a distribuição dos resíduos padronizados, sendo que se aproximaram da distribuição normal. Essa suposição foi também confirmada pelo gráfico P-P normal de regressão dos resíduos. Finalmente, o gráfico de dispersão entre os resíduos padronizados e previstos não mostrou que eles estavam aleatoriamente distribuídos; portanto, o modelo desenvolvido apresenta bom ajuste.

## DISCUSSÃO

Os indivíduos em início de TARV desta coorte apresentaram incremento na QV, sendo que possuir crença e morar com outras pessoas associaram-se positivamente à QV, enquanto possuir sinais ou sintomas de ansiedade e depressão, alguma vez ou sempre, e RAM adicional reportada foram preditores associados a pior QV.

As características dessa população foram semelhantes às de outros estudos^11 , 12^ e boletins epidemiológicos^13 , 14^ , com predominância de indivíduos do sexo masculino, jovens, cor de pele autodeclarada não branca e com alta escolaridade.

O presente estudo mostrou que, ao longo do tempo, indivíduos em uso inicial de TARV apresentaram aumento no escore global de QV, consonante com pesquisas anteriores^15^ .

A maior diferença média de QV observada no domínio físico, no qual são avaliados aspectos como dor, desconforto, energia, sono e repouso, pode estar relacionada ao uso de TARV. Esta provoca alterações no curso da infecção, como desaceleração da progressão da imunodeficiência provocada pelo vírus HIV e restauração da resposta imunológica dos indivíduos – consequentemente, pode impactar positivamente na vida das pessoas^18^ .

No domínio relações sociais, em que foram avaliadas questões relacionadas a aceitação do diagnóstico, apoio familiar, relacionamento interpessoal e vida sexual, foi observada menor diferença média de QV. A relação social é um complicador para as PVHIV, uma vez que a infecção pelo vírus pode ser uma condição de saúde estigmatizante, que envolve sentimentos de discriminação e, consequentemente, falta de apoio social e sentimentos de solidão. Considerando que esses fatores dificilmente serão influenciados pelo uso da TARV, é importante ressaltar a necessidade de melhorar as relações pessoais e o suporte social para esses indivíduos^15^ .

Foi demonstrado aumento estatisticamente significante no componente de percepção global da QV. Os benefícios dos medicamentos na QV podem ser explicados pela redução dos sintomas clínicos da infecção. Ou seja, isso reforça a necessidade de vinculação e retenção dos indivíduos ao serviço de saúde e adesão ao tratamento.

Alguns fatores comportamentais, sociodemográficos, clínicos e relacionados a TARV mostraram relação com a alteração na QV em indivíduos no início da TARV, sendo esses achados condizentes com a literatura científica^15^ .

Sentimentos de culpa, solidão e receio da morte, comumente reportados por PVHIV, estão relacionados ao exercício da espiritualidade e crenças religiosas^12 , 21^ , o que contribui para o aumento da QV e da saúde do indivíduo. Fé e crenças religiosas são importantes estratégias de enfrentamento de condições de saúde como o HIV, quando aliadas a TARV^11 , 21^ .

Nossos resultados destacam a importância do envolvimento familiar para a redução do estigma e do preconceito relacionados ao HIV. Em outro estudo utilizando o mesmo instrumento, porém com delineamento transversal^22^ , realizado na região Sul do Brasil, foi demonstrado que os indivíduos que moravam sozinhos apresentaram 30% de propensão a pior QV no domínio relações sociais. Um menor suporte emocional durante o tratamento poderia explicar parcialmente os resultados encontrados. Outro estudo transversal, realizado com 100 PVHIV no Nepal, utilizando o instrumento WHOQOL- *bref* , demonstrou que um bom apoio familiar tem maior impacto no incremento de QV, sendo estatisticamente associado à QV geral^23^ .

Sinais ou sintomas de ansiedade e depressão são fatores que podem limitar a QV de PVHIV^24^ . Existem evidências de que depressão e ansiedade são mais frequentes em PVHIV do que na população em geral^25^ , o que pode exercer influência negativa no comportamento do indivíduo e contribuir para dificuldades em relação ao tratamento e ainda para pior adesão a TARV^26^ . O impacto de sintomas psiquiátricos na QV de PVHIV também acontece devido a uma deterioração do sistema imunológico e consequente aumento da progressão da doença, justificados pelo nível aumentado de estresse e sintomas depressivos^27^ . Apesar de a HADS não ser utilizada com fins diagnósticos, e sim para avaliar sinais e sintomas de ansiedade e depressão, foi observada associação entre esses sinais e sintomas e a redução da QV nas PVHIV nas entrevistas de acompanhamento, o que evidencia a importância do acompanhamento por um profissional de saúde (psicólogo, psiquiatra, entre outros) nos serviços onde esses indivíduos estão sendo acompanhados. Em outro estudo sobre QV do grupo Ecoart, de delineamento transversal, realizado com 366 indivíduos em Belo Horizonte, estiveram associados a menor QV indivíduos solteiros, com outras comorbidades, com menor nível educacional, tabagistas, com sinais e sintomas de ansiedade e depressão^4^ . Em outro estudo, com delineamento transversal com PVHIV, realizado em Camboja, onde foram avaliadas 150 pessoas, os autores sugerem forte associação entre o incremento da QV avaliado pelo escore global de QV e pelos seis domínios do WHOQOLHIV- *bref* e a ausência de sinais ou sintomas de ansiedade e depressão^28^ .

Neste estudo, observou-se que a cada RAM adicional relatada por indivíduos em tratamento houve maior redução na QV, ou seja, quanto maior é o número de RAM relatadas, maior é a redução na QV. Este resultado é similar ao de outro estudo realizado no Brasil, que descreveu o menor número de RAM a TARV como um dos fatores associados à melhora da QV^29^ . A ocorrência de RAM pode afetar física e psicologicamente a realização de atividades profissionais e pessoais e, consequentemente, a QV de indivíduos em tratamento^5^ . A monitorização do uso de antirretrovirais é importante ferramenta para avaliar a segurança desses medicamentos na prática clínica^5^ e garantir melhor assistência e satisfação dos indivíduos com o tratamento^29^ .

Ao compararmos os indivíduos em uso do esquema TDF/3TC + DTG com aqueles em uso de TDF/3TC/EFV ou outros esquemas, não foram observadas diferenças estatisticamente significantes na diferença média de QV geral, o que pode ser explicado por parte da população do estudo Ecoart ter iniciado o uso dos ARV mesmo assintomático, em função da mudança do protocolo de tratamento de HIV pelo MS. Portanto, esses indivíduos podem não ter reconhecido a melhora na QV com o uso da TARV, independentemente do esquema utilizado. Este estudo apresenta como limitação a incompletude dos dados do Sistema de Controle de Exames Laboratoriais (Siscel), como contagem de linfócitos TCD4+ e CV. Para minimizar essa limitação, consideramos os dados do período de três meses anteriores e posteriores à segunda entrevista de acompanhamento, resultando em baixo percentual de dados faltantes.

Como pontos positivos, deve-se ressaltar que o estudo é longitudinal, que a coleta de dados foi realizada com rigor metodológico e em locais representativos da PVHIV de Belo Horizonte, o número elevado da amostra e a robustez do modelo final obtido por meio de análise multivariável. Além disso, o WHOQOLHIV- *bref* é um instrumento que avalia a QV nas duas semanas anteriores à entrevista; assim, potenciais vieses de memória foram minimizados.

Novos estudos em PVHIV com maior tempo de seguimento, utilizando o WHOQOLHIV- *bref* , bem como outros instrumentos, são necessários para mensurar as alterações na QV desses indivíduos e contribuir para o direcionamento de ações e intervenções dos profissionais de saúde que podem contribuir para aumentar a QV em PVHIV.

Conclui-se que, ao longo do tempo, PVHIV iniciando a TARV apresentaram melhora na QV. Há necessidade de acompanhar e prover cuidados de saúde, em especial para indivíduos com sinais e sintomas de ansiedade e depressão e que relatam reações adversas no início do tratamento.

Este estudo pode contribuir para o planejamento e o direcionamento de ações e políticas públicas, bem como para identificar fatores modificáveis que possam aumentar a QV desses indivíduos.
